# Mississippi Valley Dental Association

**Published:** 1872-05

**Authors:** 


### Mississippi Valley Dental Association.

THE late meeting of the Mississippi Valley Association, was in many respects, the most pleasant and successful since its organization.

The subjects proposed for discussion were such as to do credit to the Executive Committee, and command the attention of the members, and exite in them generally an earnest activity in their consideration, seldom manifest on former occasions, and the quiet, decorous attention of the "lay members" who generally attended to the business in hand, instead of whispering in knots of two or three, was a marked improvement on former occasions.

The discussions on former occasions have largely contributed to bring about a stage in the department of Operative Dentistry, so far advanced toward perfection, that it is now no very astonishing feat to fill cavities of decay, so well that the teeth thus filled, will last longer than teeth in the same mouth that were free from decay when the fillings were put in, or where new cavities are formed in the same tooth, that will occasion its loss, while the cavity that was filled remains unchanged. The loss of such teeth is of course found in the skirts, or on the hands of the patient, and not of the dentist; in a word there are hundreds of dentists all through the country who make it a common every day business, to fill decayed teeth in such a manner that the filling is more durable than the tooth in which it is placed. This would naturally suggest the necessity of looking for means and methods of imparting a better constitution to the dental organs, thus to secure a more reliable foundation on which to build those shining structures, that so many delight to rear on a basis of carious teeth. To me it appears to be quite in order that the profession should make an advance step in this direction, and toward the cultivation of a higher field of conservative dentistry.

In the department of dental appliances, the number presented was not large. Dr. Carpenter's pneumatic engine, improved to the last degree of artistic beauty and mechanical excellence, constitutes an admirable adjunct to a complete dental operating room. Its complexity however is such as to render it worthless in the hands of one of slovenly habits and unskilled in mechanical appliances, but for the fact that its neatness and accuracy of arrangement and adjustment, and the fidelity and skill manifested throughout all the details of its arrangement and construction, is such as to furnish a strong guarantee against any liability to derangement.

The appliance presented by Dr. Baxter, is one of the neatest "tricks" for concentrating the optical perspicacity, or directing a ray of concentrated light on a given point. For simplicity of construction and convenience of application, it has no superior, but it needs no commendation; "it speaks for itself."

There is one thought connected with this matter of concentration, that claims a passing notice, viz: the concentration of a purpose, the will, the soul, on the accomplishment of a specified object.

Every one present was charmed with the beautiful representations submitted by Dr. C. Palmer.

The secret of Dr. Palmer's marvellous success, is comprehended in his power of concentrating his attention on a given point, and concentrating his effort on the accomplishment of a given purpose.

The Dr. is not a brilliant man in the common acceptation of the term, and his startling achievements are but the result of patient plodding effort, of indomitable and untiring industry, whether he possesses this faculty as a free gift from dame nature, or whether he holds his power of self-control, as an acquisition, won by the same direct and determined effort, it is plain that his beautiful productions had their origin in this peculiar mental attribute.

There are members in the professson who possess a heterogenous jumble of fragmentary facts and ideas in ample profusion, greater in the aggregate than Dr. Palmer can justly claim, and who are still utterly inefficient, from a lack of self-control, of steady and well-directed effort.

If some teacher should arise in our midst, who could enlighten and inspire the public mind in this direction, he might thus confer untold benefits on us one and all. Mc.

